# Supplementary material for: Viral expansion after transfer is a primary driver of influenza A virus transmission bottlenecks
Source: PLoS Biol. 2025 Sep 2;23(9):e3003352. doi: 10.1371/journal.pbio.3003352 (PMC12413080; doi:10.1371/journal.pbio.3003352)
Supplement: S1 Fig — A) Schematic showing transmission set-up in guinea pigs. For each of the three experimental replicates, eight guinea pigs were intranasally inoculated with 5 × 10^4^ PFU of Pan/99 NA-BC in 300 µL. Twenty-four hours post-inoculation, a single naïve animal was placed with each inoculated animal. Cages either allowed for direct contact (n = 4) or maintained separation with a double-walled, perforated metal barrier (n = 4). B) Viral titers in nasal lavage samples in direct contact and aerosol exposure settings from replicates 1, 2, and 3. Inoculated animals are shown in blue and exposed animals in red (direct contact) or yellow (aerosol contact). The dashed black line represents the limit of detection (50 PFU/mL). Paired animals share the same line type. Negative results are plotted at the limit of detection. S1A Fig created in BioRender. Underlying data are available in S2 Data. (PDF) [file pbio.3003352.s001.pdf]

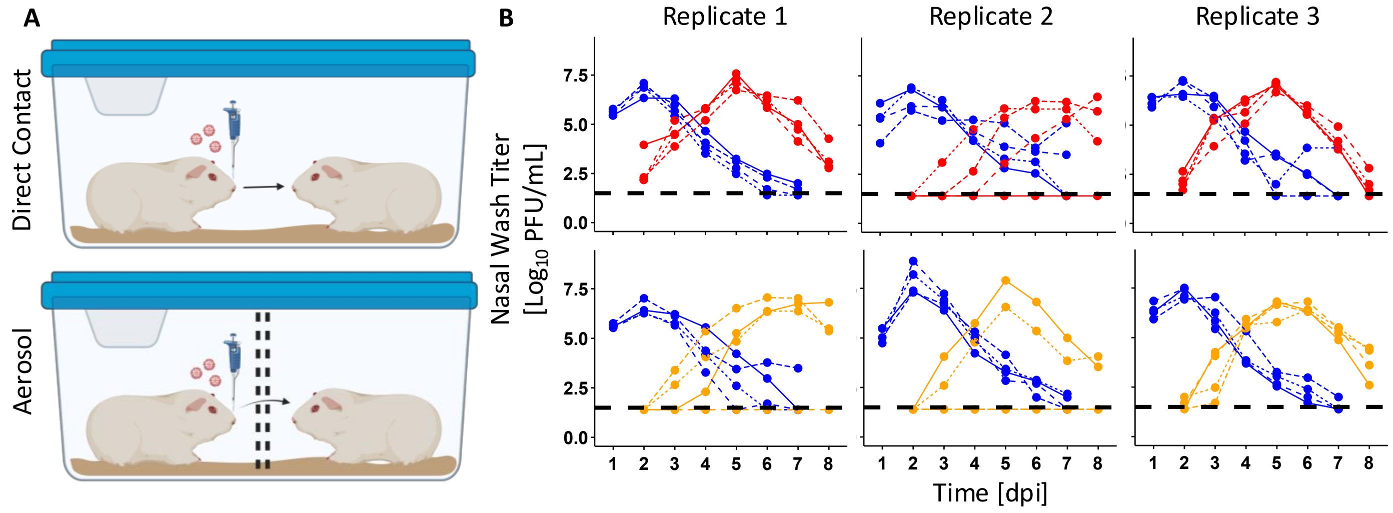

Supplemental Figure 1. Pan/99 NA-BC infects inoculated animals and transmits to exposed animals. A) Schematic showing transmission set-up in guinea pigs. For each of the three experimental replicates, eight guinea pigs were intranasally inoculated with 5 x 10^4 PFU of Pan/99 NA-BC in 300 **µ**L. Twenty-four hours post-inoculation, a single naïve animal was placed with each inoculated animal. Cages either allowed for direct contact (n = 4) or maintained separation with a double-walled, perforated metal barrier (n = 4). **B)** Viral titers in nasal lavage samples in direct contact and aerosol exposure settings from replicates 1, 2, and 3. Inoculated animals are shown in blue and exposed animals in red (direct contact) or yellow (aerosol contact). The dashed black line represents the limit of detection (50 PFU/mL). Paired animals share the same line type. Negative results are plotted at the limit of detection. Supplemental Figure 1A created in BioRender. Underlying data are available in S2 Data.
