# Supplementary material for: Viral expansion after transfer is a primary driver of influenza A virus transmission bottlenecks
Source: PLoS Biol. 2025 Sep 2;23(9):e3003352. doi: 10.1371/journal.pbio.3003352 (PMC12413080; doi:10.1371/journal.pbio.3003352)
Supplement: S2 Fig — Data from experimental replicates 1, 2, and 3 are shown in panels A, B, and C, respectively. Guinea pig ID numbers are shown in the upper right corner of each plot. Nasal lavage titers are indicated by the total height of each bar. Colors within the bars represent unique barcodes, and the height of each color indicates the relative frequency within the sample. Only samples that were plaque-positive are shown. Red lines show LOD of 50 PFU/mL. Plots for individual animals are paired with those of their cage mate. For the exposed animal in the first aerosol transmission pair of Replicate 1 (GP10), most reads were discarded due to poor quality, and the data from this animal were excluded from further analyses. Data underlying this figure are available in S2 Data and at https://doi.org/10.5281/zenodo.16115331. (PDF) [file pbio.3003352.s002.pdf]

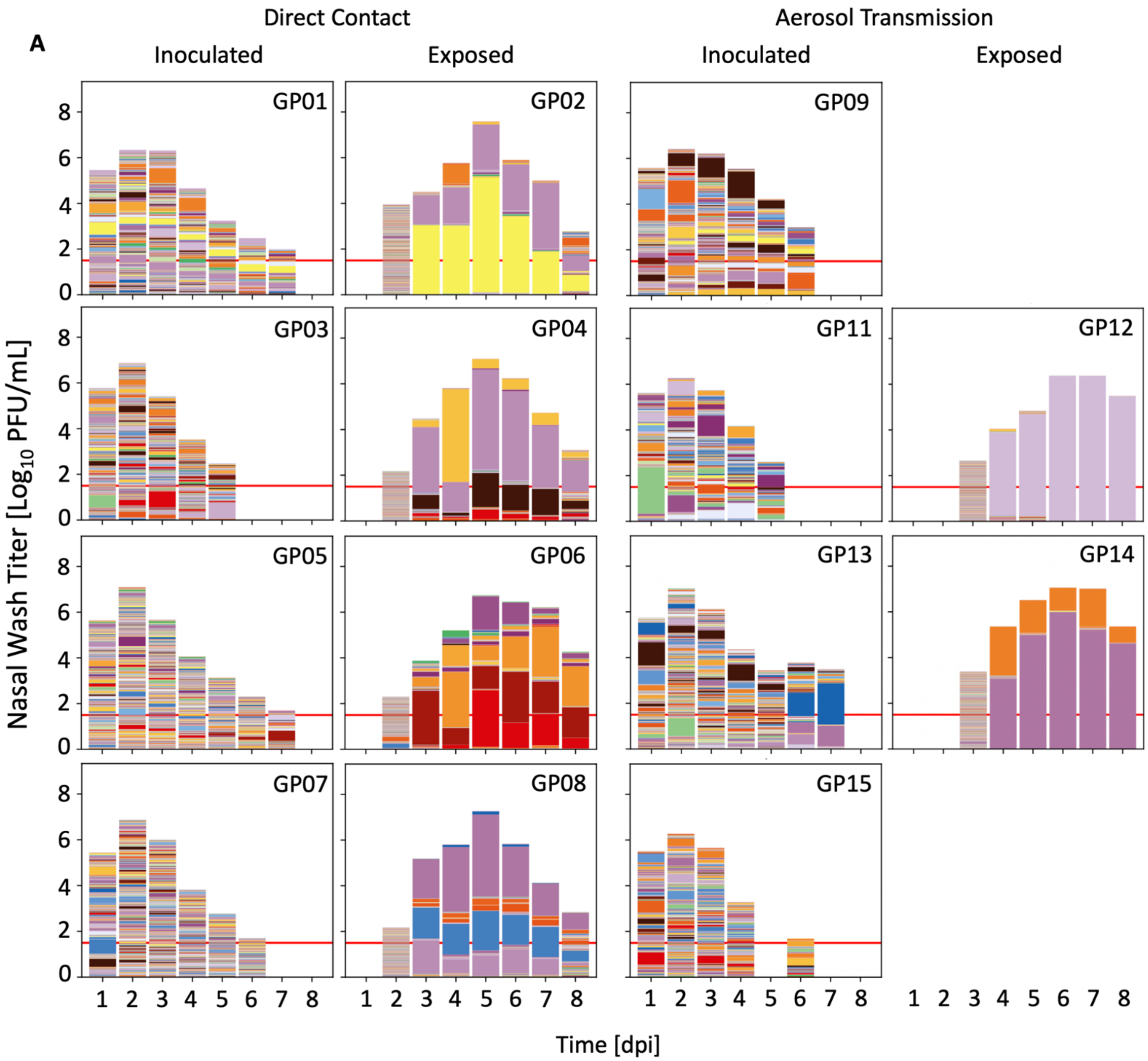
A
Direct Contact
Aerosol Transmission
Inoculated
Exposed
Inoculated
Exposed
GP01
GP02
GP09
GP03
GP04
GP11
GP12
GP05
GP06
GP13
GP14
GP07
GP08
GP15
Nasal Wash Titer [Log10 PFU/mL]
Time [dpi]

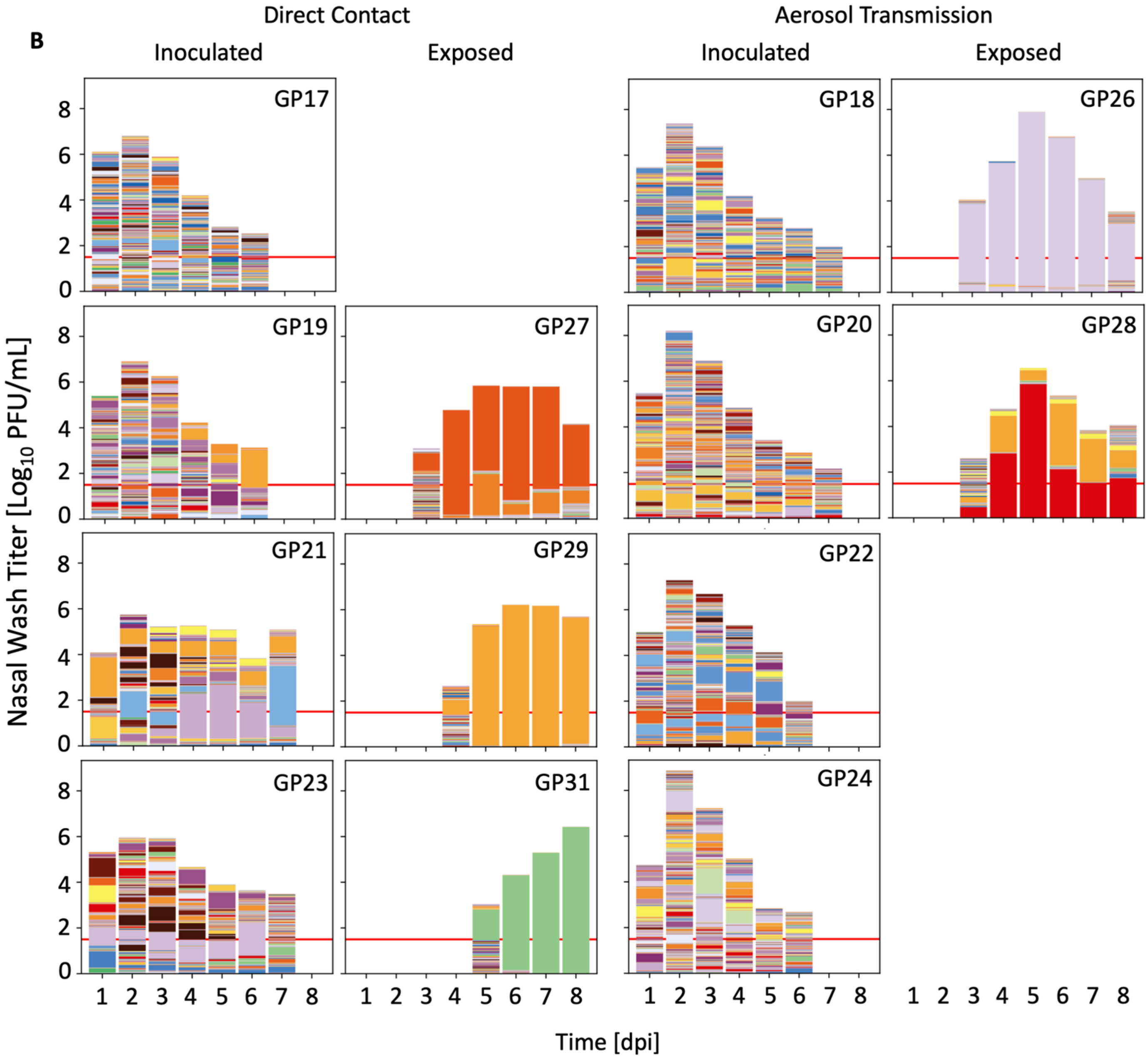

B
Direct Contact
Aerosol Transmission
Inoculated
Exposed
Inoculated
Exposed
GP17
GP18
GP26
GP19
GP27
GP20
GP28
GP21
GP29
GP22
GP23
GP31
GP24
0 2 4 6 8
1 2 3 4 5 6 7 8
Nasal Wash Titer [Log10 PFU/mL]
Time [dpi]

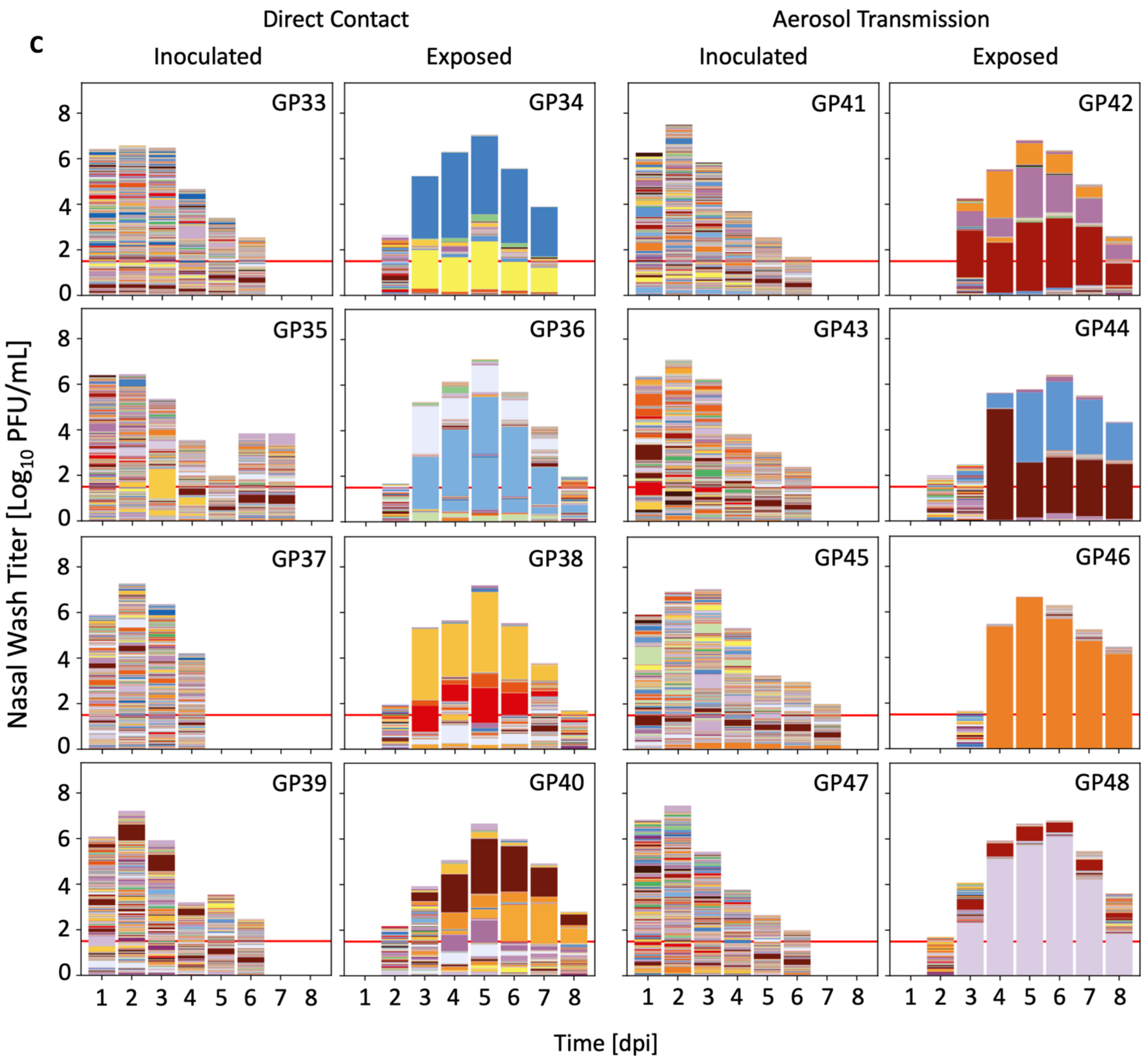

Supplemental Figure 2. Population diversity declines between inoculated and exposed guinea pigs. Data from experimental replicates 1, 2, and 3 are shown in panels **A, B,** and **C,** respectively. Guinea pig ID numbers are shown in the upper right corner of each plot. Nasal lavage titers are indicated by the total height of each bar. Colors within the bars represent unique barcodes, and the height of each color indicates the relative frequency within the sample. Only samples that were plaque-positive are shown. Red lines show LOD of 50 PFU/mL. Plots for individual animals are paired with those of their cage mate. For the exposed animal in the first aerosol transmission pair of Replicate 1 (GP10), most reads were discarded due to poor quality, and the data from this animal were excluded from further analyses. Data underlying this figure are available in S2 Data and at https://doi.org/10.5281/zenodo.16115331.
