# Supplementary material for: Viral expansion after transfer is a primary driver of influenza A virus transmission bottlenecks
Source: PLoS Biol. 2025 Sep 2;23(9):e3003352. doi: 10.1371/journal.pbio.3003352 (PMC12413080; doi:10.1371/journal.pbio.3003352)
Supplement: S3 Fig — Shannon diversity (left), richness (right, left axis), and evenness (right, right axis) were determined for inoculated animals (i, blue) and exposed animals (e, red) in replicate experiments 1, 2, and 3 (A, B, and C, respectively). Data from cage mates are paired. In the right-hand plots, bold colors show richness and faded colors show evenness. Line widths show 95% confidence intervals based upon subsampling of barcode reads. Underlying data available in S2 Data. (PDF) [file pbio.3003352.s003.pdf]

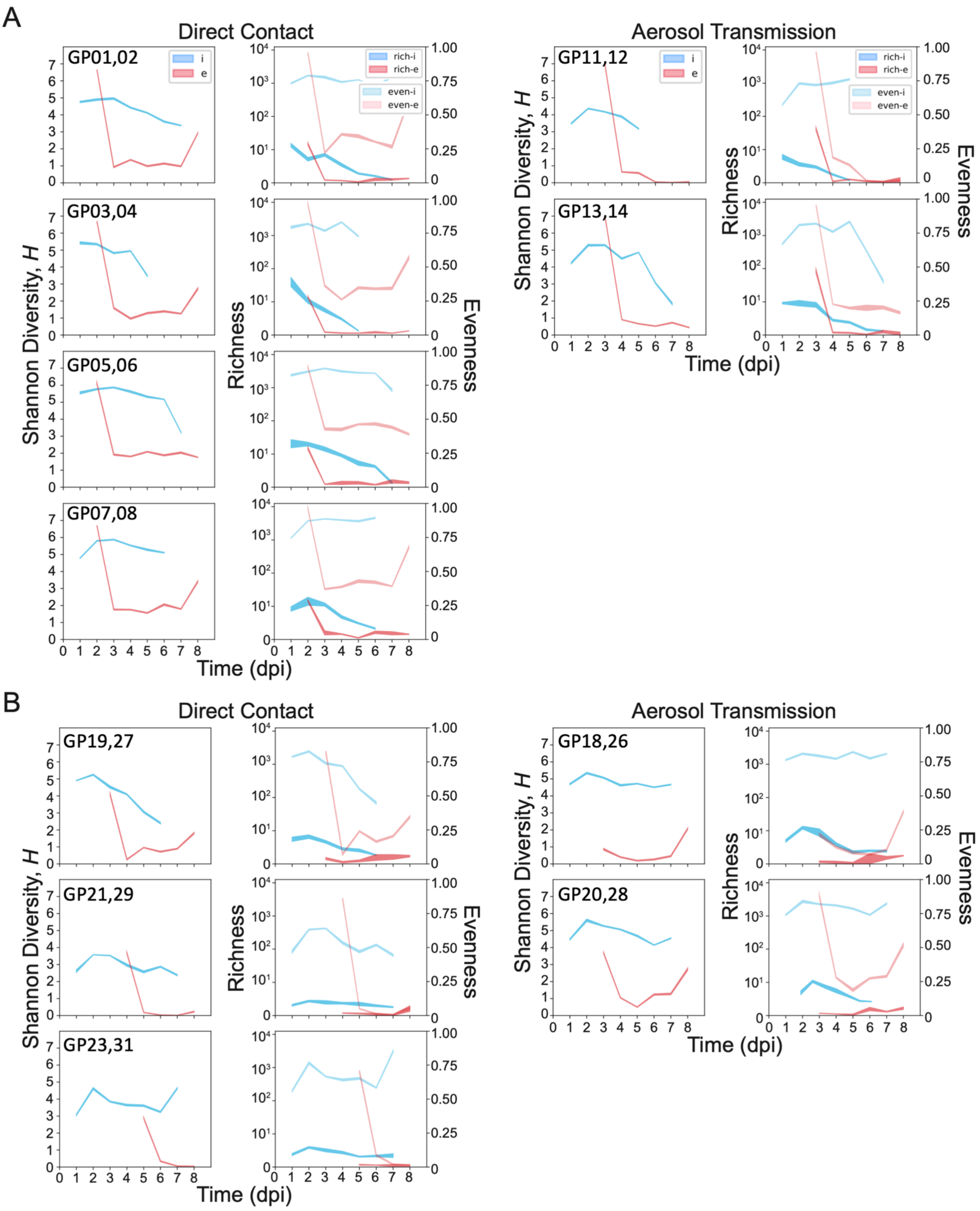

A
Direct Contact
Aerosol Transmission
GP01,02
GP03,04
GP05,06
GP07,08
GP11,12
GP13,14
i
e
rich-i
rich-e
even-i
even-e
Shannon Diversity, H
Richness
Evenness
Time (dpi)
B
Direct Contact
Aerosol Transmission
GP19,27
GP21,29
GP23,31
GP18,26
GP20,28
Shannon Diversity, H
Richness
Evenness
Time (dpi)

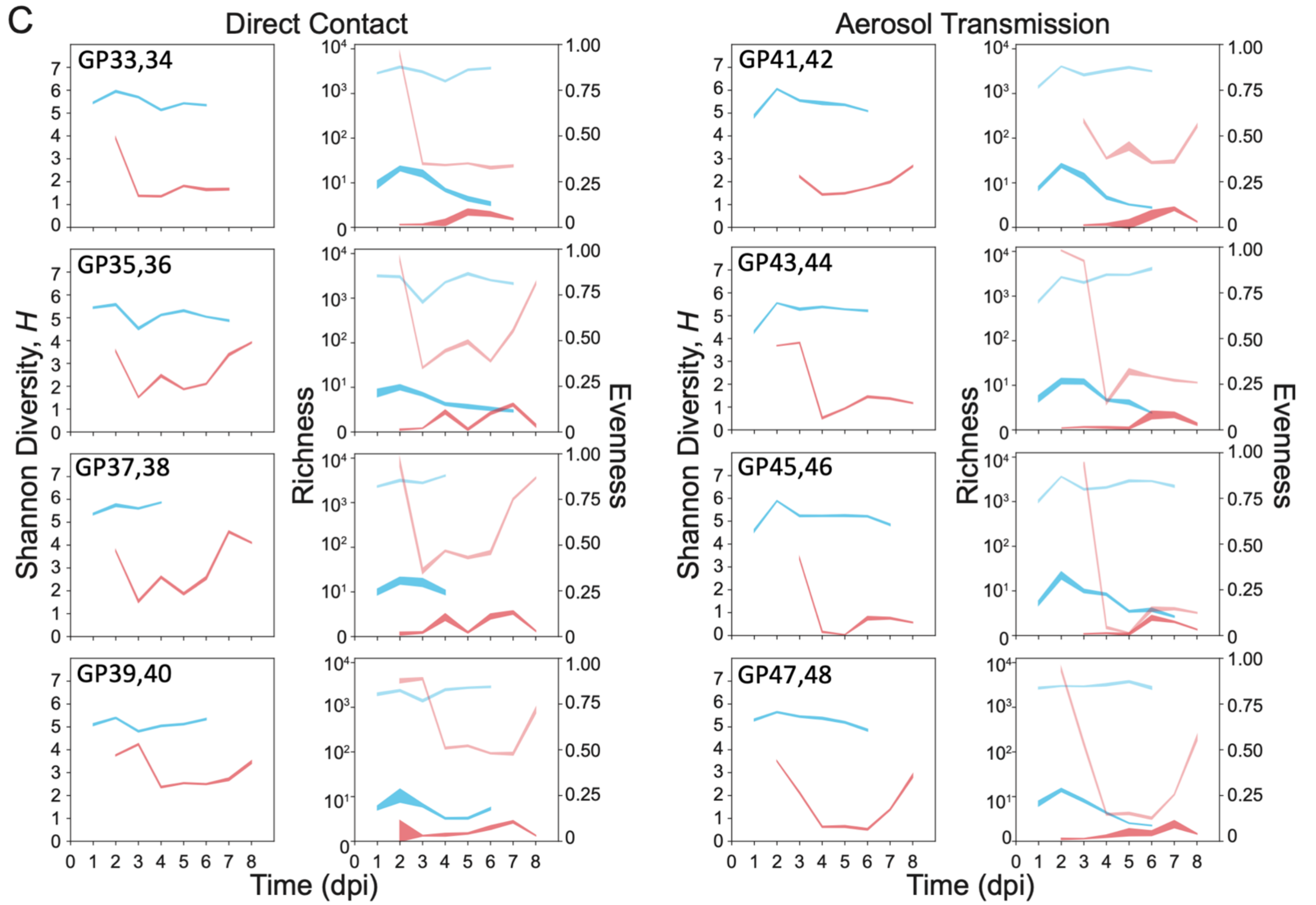

Supplemental Figure 3. Changes in richness and evenness both contribute to alterations in diversity. Shannon diversity (left), richness (right, left axis), and evenness (right, right axis) were determined for inoculated animals (i, blue) and exposed animals (e, red) in replicate experiments 1, 2, and 3 (**A, B,** and **C**, respectively). Data from cage mates are paired. In the right-hand plots, bold colors show richness and faded colors show evenness. Line widths show 95% confidence intervals based upon subsampling of barcode reads. Underlying data available in S2 Data.
