# Supplementary figures and images for: Viral expansion after transfer is a primary driver of influenza A virus transmission bottlenecks
Source: PLoS Biol. 2025 Sep 2;23(9):e3003352. doi: 10.1371/journal.pbio.3003352 (PMC12413080; doi:10.1371/journal.pbio.3003352)

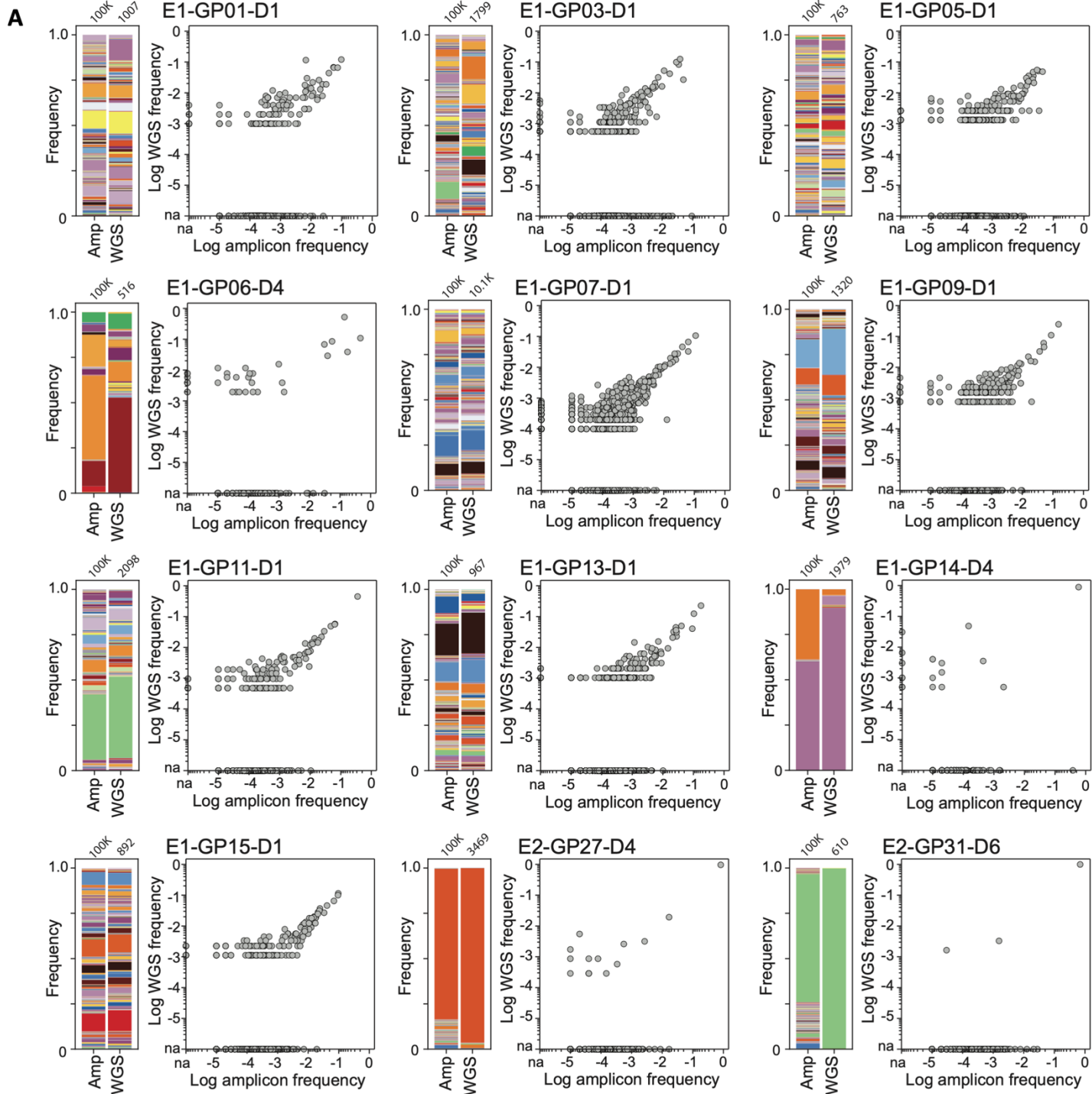

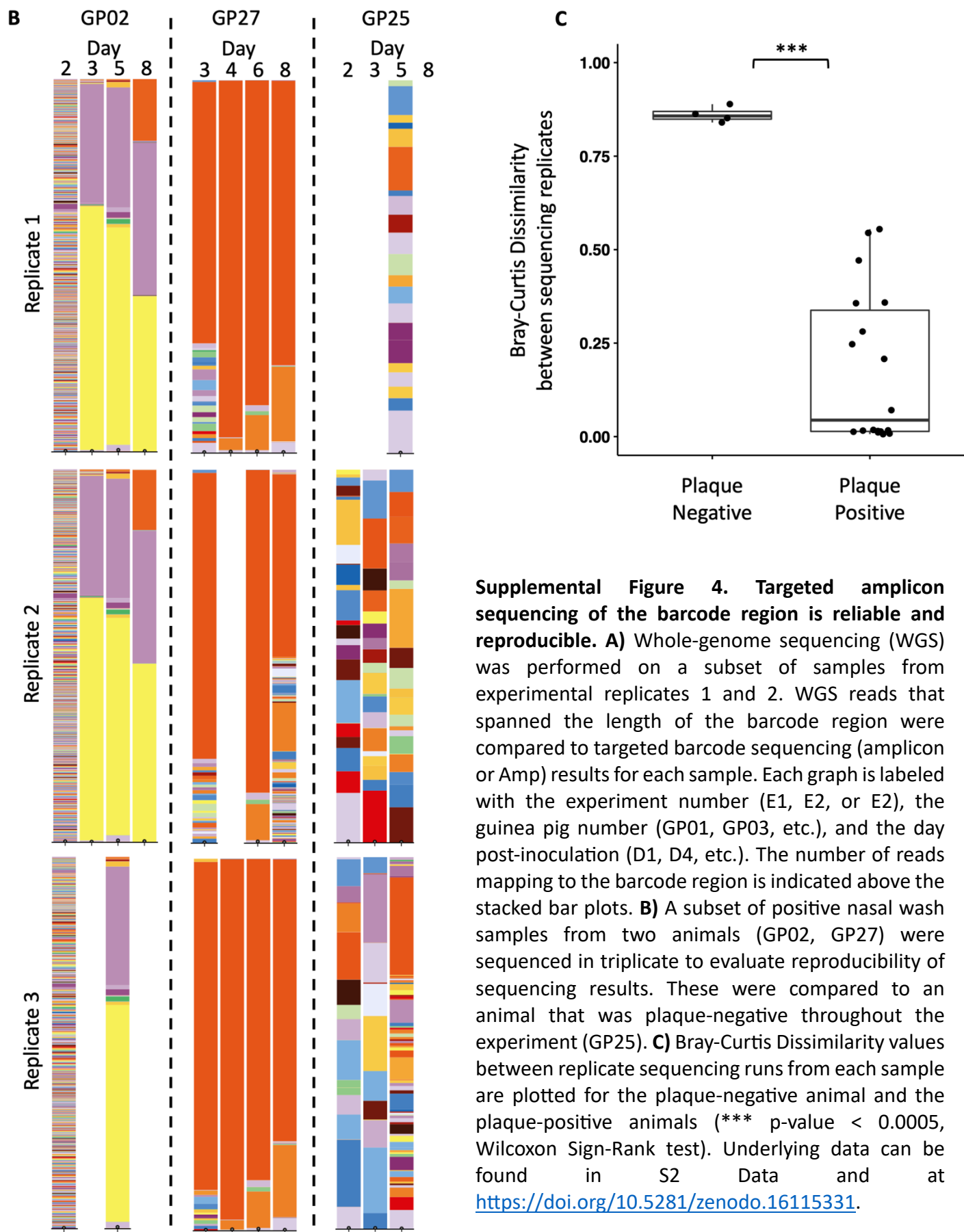

Supplement: S4 Fig — A) Whole-genome sequencing (WGS) was performed on a subset of samples from experimental replicates 1 and 2. WGS reads that spanned the length of the barcode region were compared to targeted barcode sequencing (amplicon or Amp) results for each sample. Each graph is labeled with the experiment number (E1, E2, or E2), the guinea pig number (GP01, GP03, etc.), and the day post-inoculation (D1, D4, etc.). The number of reads mapping to the barcode region is indicated above the stacked bar plots. B) A subset of positive nasal wash samples from two animals (GP02, GP27) were sequenced in triplicate to evaluate reproducibility of sequencing results. These were compared to an animal that was plaque-negative throughout the experiment (GP25). C) Bray-Curtis Dissimilarity values between replicate sequencing runs from each sample are plotted for the plaque-negative animal and the plaque-positive animals (*** p-value < 0.0005, Wilcoxon Sign-Rank test). Underlying data can be found in S2 Data and at https://doi.org/10.5281/zenodo.16115331. (PDF) [file pbio.3003352.s004.pdf]
