# Supplementary material for: Viral expansion after transfer is a primary driver of influenza A virus transmission bottlenecks
Source: PLoS Biol. 2025 Sep 2;23(9):e3003352. doi: 10.1371/journal.pbio.3003352 (PMC12413080; doi:10.1371/journal.pbio.3003352)
Supplement: S5 Fig — At each inoculation dose indicated above the plots, three guinea pigs were inoculated intranasally with Pan/99 NA-BC. A) Nasal lavage titers and barcode compositions over time. The height of the bars indicates viral titer in samples above the limit of detection (50 PFU/mL). Colors indicate barcodes detected, with barcode frequency shown by the height of the color. B) Maximum measured Shannon diversity, richness, and evenness of barcode compositions in each animal are plotted by inoculation dose (blue). For comparison, theoretical data are plotted to show the expected characteristics of inocula of each size derived from a perfectly even population of 4,096 barcodes (red) and from the Pan/99 NA-BC passage 1 stock (orange). Ninety-five% confidence intervals are shown with shading. Statistical significance was determined at each inoculation dose by Kruskal–Wallis Test (* p-value < 0.01, ** p-value < 0.001) comparing the medians of the experimental data with those of the perfectly even population and that of the passage 1 viral stock. Underlying data can be found in S2 Data and at https://doi.org/10.5281/zenodo.16115331. (PDF) [file pbio.3003352.s005.pdf]

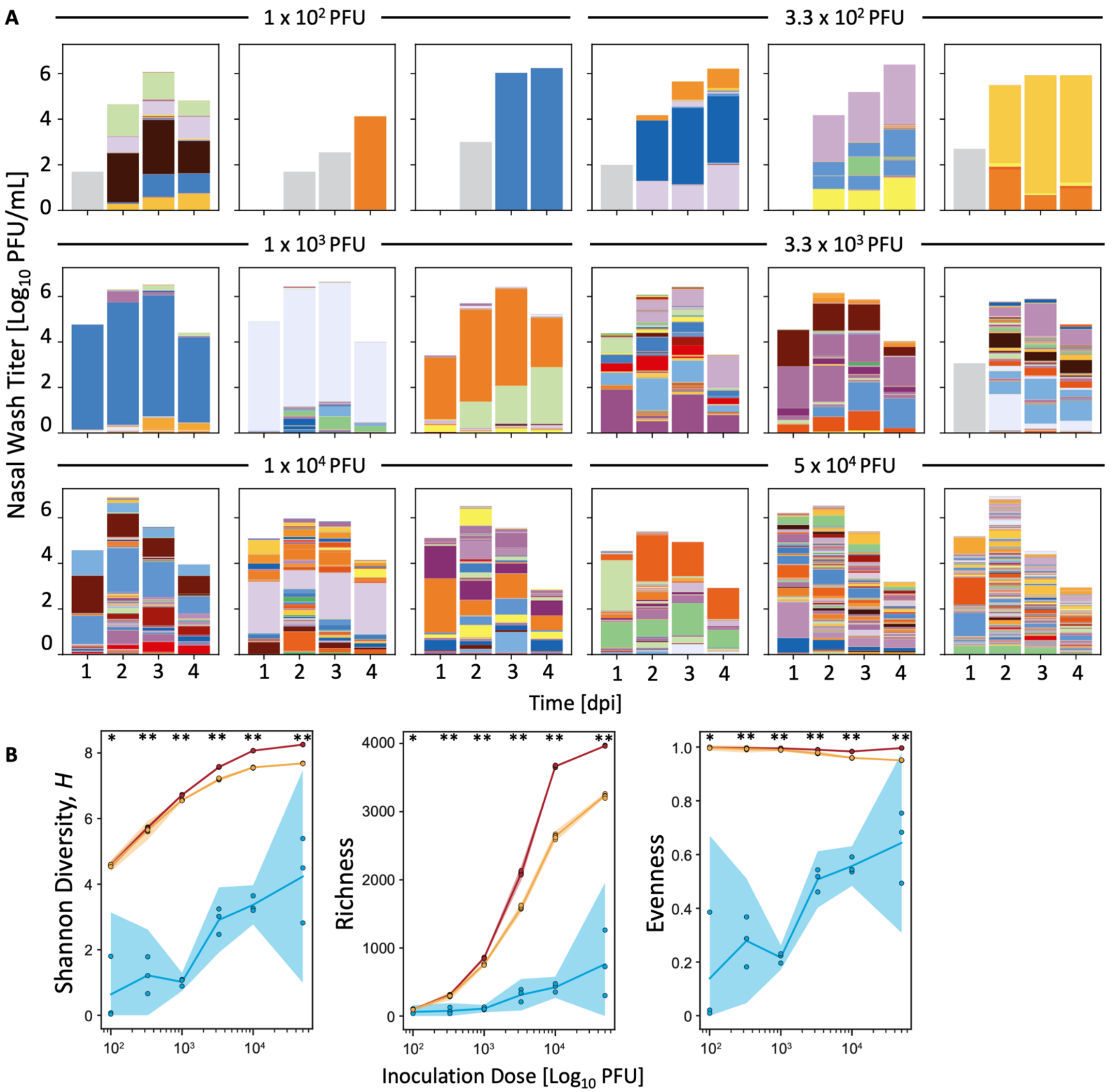

Supplemental Figure 5. Growth-induced bottlenecks are not detected in inoculated animals, even at low inoculation doses. At each inoculation dose indicated above the plots, three guinea pigs were inoculated intranasally with Pan/99 NA-BC. **A)** Nasal lavage titers and barcode compositions over time. The height of the bars indicates viral titer in samples above the limit of detection (50 PFU/mL). Colors indicate barcodes detected, with barcode frequency shown by the height of the color. **B)** Maximum measured Shannon diversity, richness, and evenness of barcode compositions in each animal are plotted by inoculation dose (blue). For comparison, theoretical data are plotted to show the expected characteristics of inocula of each size derived from a perfectly even population of 4096 barcodes (red) and from the Pan/99 NA-BC passage 1 stock (orange). 95% confidence intervals are shown with shading. Statistical significance was determined at each inoculation dose by Kruskal-Wallis Test (* p-value < 0.01, ** p-value < 0.001) comparing the medians of the experimental data with those of the perfectly even population and that of the passage 1 viral stock. Underlying data can be found in S2 Data and at https://doi.org/10.5281/zenodo.16115331.
